# A decade of road traffic fatalities among the elderly in north-West Iran

**DOI:** 10.1186/s12889-017-4976-2

**Published:** 2018-01-08

**Authors:** Homayoun Sadeghi-Bazargani, Bahram Samadirad, Farnaz Moslemi

**Affiliations:** 1 Road Traffic Injury Research Center, Tabriz University of Medical Sciences, Tabriz, Iran; 2 Legal Medicine Research Center, Forensic Medicine Organization, Tehran, Iran; 3 Department of Anesthesiology, Alzahra University Hospital, Tabriz University of Medical Sciences, Tabriz, Iran

**Keywords:** Elderly, Injuries, Mortality, Road traffic accidents, Head trauma

## Abstract

**Background:**

Iran has a uniquely catastrophic status for road traffic injury incidence and fatality. The elderly account for a substantial number of the hospitalizations and fatalities due to traffic injuries. The aim of this study was to investigate the crash mechanisms and medical outcomes of traffic fatalities among the elderly in East Azerbaijan province of Iran during the period 2006–2016.

**Methods:**

A total of 9435 fatalities registered in East Azerbaijan forensic medicine database, Iran, during 2006–2016, were investigated. Of these, 1357 were elderly persons (age > 65). Both victim- and crash-related variables were compared for the elderly and other age groups. Bivariate and multivariate analysis methods were applied using Stata statistical software package version 13.

**Results:**

Of the 9435 fatalities, 1357 victims (14.4%) were elderly persons. The mean age of the elderly traffic fatalities was 75.3(SD = 6.2) years. About 78% of the elderly versus 80% of those in other age groups were males. A decreasing trend of fatal traffic accidents was observed over the study period both for the elderly and other age groups. The elderly were nearly seven times more likely to die as a pedestrian compared to other age groups. By exclusively analyzing pedestrians, it was found that motorcycles were responsible for pedestrian deaths in 9.1% of the fatalities while this figure was 5.5% for pedestrians in other age groups killed in a traffic accident (*P* < 0.05). About 56% (*N* = 761) of the elderly died in hospital which was higher than the proportion for other age groups (39%). Ambulance was the main vehicle for transferring the injured victims in four-fifths of the cases both for the elderly and other age groups. Although, in the present study, head injuries were the most common type of injury regardless of the age group, the elderly had a lower percentage of head injuries and a higher percentage of injuries to the torso, pelvis and limbs compared to younger victims.

**Conclusions:**

Pedestrian inner-city crashes in East Azerbaijan province of Iran are a major cause of road injury fatalities among the elderly and should be considered as a priority in road safety interventions.

## Background

Global figures show nine deaths per 10,000 motor vehicles, while Iran’s statistics confirmed 37 deaths per 10,000 motor vehicles, more than four times the universal average, due to road traffic injuries (RTIs) [1]. As a consequence, the conditions of RTIs in Iran were put on the critical list by the World Bank [1]. Iran has a uniquely catastrophic status for road traffic injury incidence and fatality [2].

It is estimated that the proportion of the world’s elderly population will double from about 11% in 2000 to 22% in 2050 [3]. The proportion of elderly people in the population is increasing significantly in many low- and middle-income countries (LMICs). A substantial proportion of the elderly suffer from hearing loss, weakened vision, a decline in cognitive function, and musculoskeletal problems, making them prone to traffic accidents [4]. The elderly account for a substantial number of the hospitalizations and fatalities due to traffic injuries. In a previous study on burden of road traffic injuries in Iran, it has been shown that younger adults had a higher risk of being involved in non-fatal crashes, the elderly had the highest total death rates, largely because of pedestrian crashes [5]. Other studies from china have also shown that the incidence and mortality rates of traffic crashes have a U-shaped distribution by driver age, such that the young and old drivers are at higher risk of dying or being injured, and the risk of fatality has fivefold increase for drivers 75–79 years of age and 3.5-fold for those 80 years and over [6, 7]. This high mortality among the elderly necessitates to pay more attention on investigating the patterns of traffic mortalities among the elderly. Regardless of the bulk of literature on the epidemiology of traffic injuries, evidence on the details of fatal injury mechanisms, along with their medical outcomes among the elderly, is rarely available especially from LMICs including Iran. The aim of this study was to investigate crash mechanisms and medical outcomes of traffic fatalities among the elderly in East Azerbaijan province of Iran through the years 2006–2016.

## Methods

The present study was conducted in East Azerbaijan Province in Iran on traffic injury fatalities among the elderly registered in the East Azerbaijan Forensic Medicine Organization, over a 10-year period. East Azerbaijan province is located in North-West Iran with a population of 3,725,000 people, according to the most recent census in 2011. All the elderly fatalities due to traffic injuries registered through the time period from March 2006 to March 2016 were investigated in this study. The elderly age group was defined as aged >65 years. The elderly fatalities were investigated while being compared with the traffic fatalities in other age groups. All the persons with an age above 65 years at the time of death, due to traffic injuries, were classified as elderly and the younger victims were classified as other age groups.

The study’s data source was the East Azerbaijan Forensic Medicine Organization which covers the whole province. Under Iran’s national legislation, all road traffic injury fatalities within 30 days after the crash must by law be investigated to establish the precise reasons for death via autopsy at the forensic medicine centers. According to Iranian regulations, the definition for a traffic crash includes any injury, fatality and damage caused by collision of vehicles with each other or collision of vehicles with animals, humans or objects. Moreover, any injury, damage or fatality caused in other situations affecting the road users including the vehicle falling from a height or rollover of the vehicle is also included. The forensic medicine centers in each district register such deaths and render all data to the central Forensic Medicine Organization located in the capital city, Tabriz.

All the 9435 fatal traffic injury cases registered in the East Azerbaijan Forensic Medicine Organization database were compiled without sampling for a 10-year period from 21 March 2006 to 20 March 2016 equal to Persian calendar years 1385–1394. 39 records (0.4%) were excluded due to incomplete information.

Several variables were measured for all the participants including; 1) crash-related data consisting of crash mechanisms, crash counterparts, inner/outer city crash and crash time, and 2) Victim-related data including demographic data, main cause of death, injured organs, place of death, and mode of transfer to hospital. Details of research methodology and data collection tools are published as the research protocol elsewhere [8]. In this study, the data for the accident victims’ mode of transport to the hospital was only available for the last seven years of study.

Data analysis was carried out using Stata version 13.1 statistical software package (StataCorp, Texas). Descriptive statistics such as frequency, relative frequency, mean, standard deviation (SD), odds ratios (OR) and 95% confidence intervals of the effect size (95% CI) were calculated. Inferential statistical methods such as Chi-squared test and multivariate logistic regression were also applied to assess potential associations between variables with a categorical scale and pre-hospital mortality, respectively. A *P*-value below 0.05 was considered as the statistical significance level through bivariate analysis and *P* < 0.1 for selecting the variables to introduce into the multivariate regression model.

## Results

A total of 9435 traffic fatalities were registered in East Azerbaijan forensic medicine database through the Persian calendar years of 1385–1394 equivalent to the time period between March 2006 and March 2016. The elderly comprised 14.4% of the casualties (1357 victims).

**Fig. 1:**
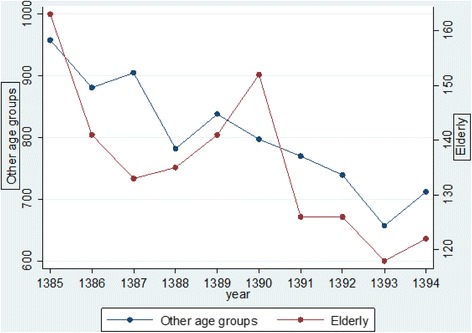
Trend of elderly fatalities due to traffic injuries in East Azerbaijan Province of Iran compared with other age groups (March 2006–March 2016)

A decreasing trend of fatal traffic accidents was observed over the study period both for the elderly and other age groups, however, a secondary peak was observed in 2011 (Fig. 1).

The mean age of the elderly traffic fatalities was 75.3 (SD = 6.2) years. Gender distribution was similar between the elderly and other age groups such that about 78% of the elderly and 80% of other age groups were males respectively (*P* = 0.15). The elderly were nearly seven times more likely to die as a pedestrian compared to other age groups, such that 821(61%) of the elderly who died due to traffic injuries were pedestrians versus 18.5% being pedestrians in other age groups (*P* < 0.001). Distribution of the fatalities according to the type of vehicle used, is compared for the elderly and other age groups in Table 1.

**Table 1:** Distribution of the traffic injury victims according to the type of vehicle used for transport comparing the elderly victims with other age groups

Type of vehicle used	Elderly fatalities	Other age fatalities	Total
No vehicle used(Pedestrian)	823 (60.7%)	1471 (18.3%)	2294 (24.4%)
Car	306 (22.6%)	3453 (4%)	3759 (40%)
Bus or minibus	31 (2.3%)	196 (2.4%)	227 (2.4%)
Pickup truck	54 (4%)	640 (8%)	694 (7.4%)
Other trucks and trailers	5 (0.37%)	276 (3.4%)	281 (3%)
Motorcycles	85 (6.3%)	1751 (21.8%)	1836 (19.5%)
Bikes	30 (2.2%)	74 (0.9%)	104 (1.1%)
Other vehicles	14 (1%)	100 (1.3%)	114 (1.2%)
Unknown or missing	9 (0.7%)	78 (1%)	87 (0.9%)
Total	1357 (100%)	8039 (100%)	9396

By exclusively analyzing pedestrians, it was found that motorcycles were responsible for pedestrian deaths in 9.1% of the fatalities while this figure was 5.5% for pedestrians in other age groups killed in a traffic accident (*P* < 0.05) and the likelihood of an elderly pedestrian dying after a motorcycle crash was nearly 1.7 times more than similar casualties in other ages (OR = 1.72; 95% CI: 1.24–2.39). About half of the elderly victims (51.1%) died due to an inner city crash, while about a quarter of the other age victims (26.3%) died after inner city crashes. About 56% (761 deaths) of the elderly died in hospital which was higher than the proportion for other age groups (39%). According to the results of logistic regression analysis, the elderly deaths were twice as likely to happen in hospital compared to fatalities in other age groups (OR = 2.1; 95% CI: 1.8–2.3). The odds ratio decreased to 1.5 (95% CI: 1.3–1.7) after adjusting for place of accident (inner city vs. outer city) and the victim’s role in the traffic (pedestrian vs. vehicle users).

The body parts injured in fatal traffic injuries compared between the elderly and other age groups are given in Table 2, showing that head trauma was the most common type of trauma among fatal traffic injuries among the elderly. Although, head injuries were the most common type of injury regardless of the age group, they had a lower percentage of head injuries and a higher percentage of injuries to the torso, pelvis and limbs when compared to younger victims.

**Table 2:** Body parts injured in fatal traffic injuries compared between the elderly and other age groups in East Azerbaijan (March 2006–March 2016)

Body organ injured	Elderly	Other ages	Total	*P*-value*
Head/Face	1048 (77.2%)	6558 (81.6%)	7606 (81%)	<0.001
Neck	78 (5.8%)	515 (6.4%)	593 (6.3%)	0.36
Chest/abdomen	550 (40.5%)	2530 (31.5%)	3080 (32.8%)	<0.001
Back trunk	34 (2.5%)	156 (1.9%)	190 (2%)	0.17
Pelvis	166 (12.2%)	625 (7.7%)	791 (8.4%)	<0.001
Upper limbs	137 (10.1%)	524 (6.5%)	661 (7%)	<0.001
Lower limbs	174 (12.8%)	748 (9.3%)	922 (9.8%)	<0.001

*P*-values are based on chi-squared test to investigate whether proportions of injury for each organ are different between the elderly and other age fatalities

**Fig. 2:**
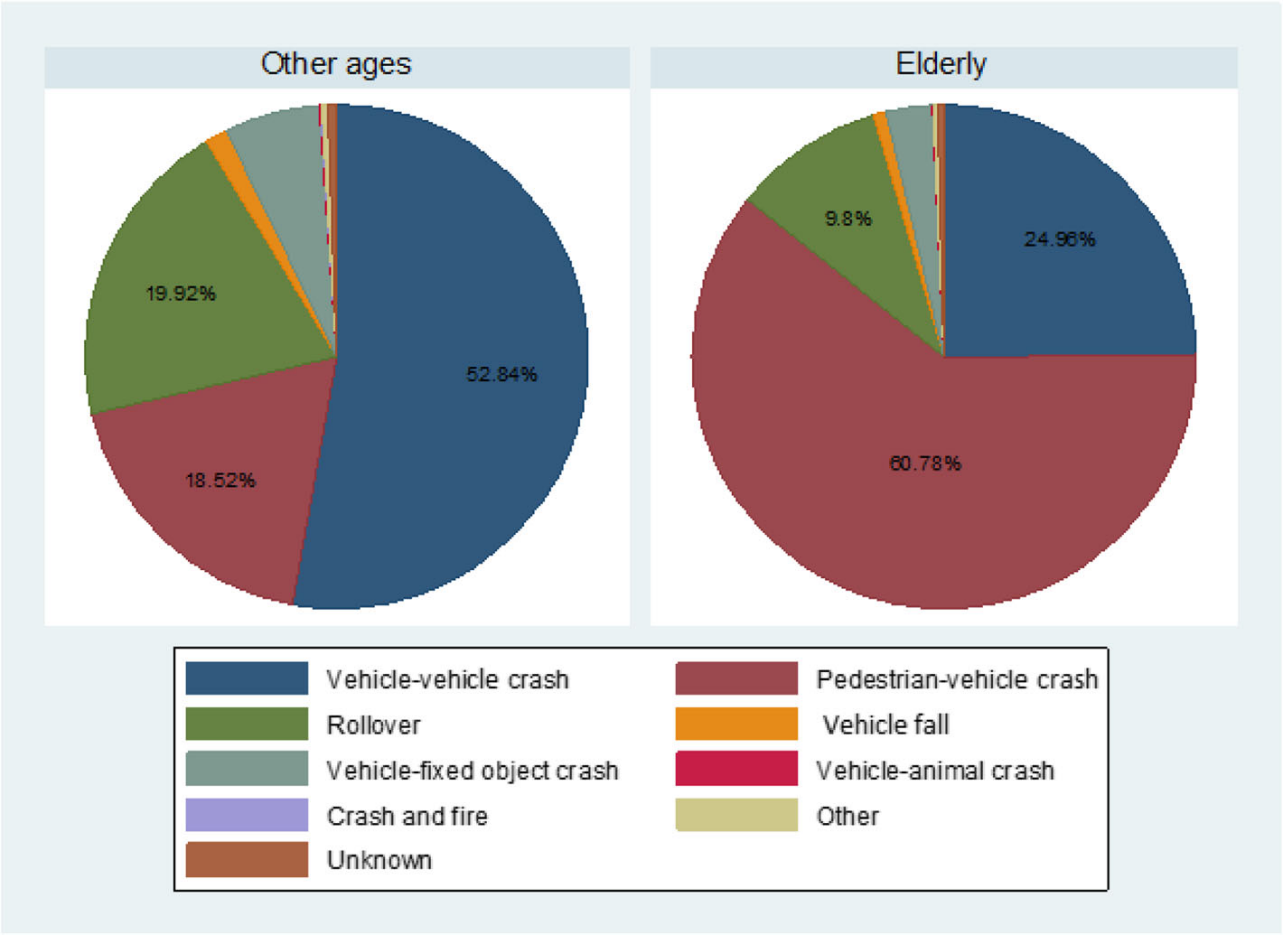
Crash mechanism in fatal traffic injuries compared for the elderly and other age groups in East Azerbaijan (March 2006–March 2016)

The crash mechanism causing death is compared for the elderly and other age groups in Fig. 2 showing that the most common crash mechanism was a vehicle-vehicle crash among other age groups in 4209 victims (52.8%) while the most common crash mechanism among the elderly was a vehicle-pedestrian crash in 823 cases (60.8%).

**Fig. 3:**
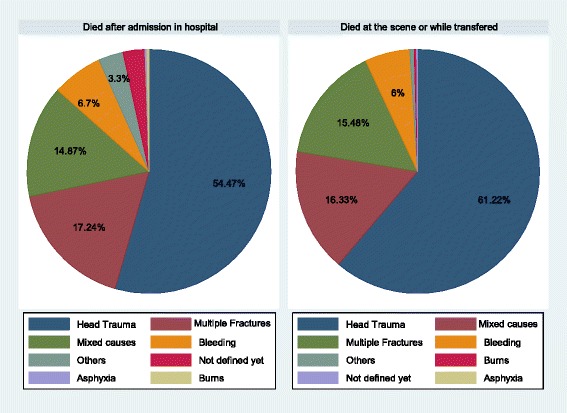
Distribution of elderly mortalities by cause of death before and after hospitalization in East Azerbaijan (March 2006–March 2016)

Head trauma and multiple fractures were the two most common causes of death among the elderly (Table 3). Among those aged over 65 who died prior to hospitalization, head trauma was the main cause of death in 61.2% (360) of the victims versus 54.5% among those who died after hospitalization (Fig. 3).

**Table 3:** Main cause of death after traffic injuries compared between the elderly and other age groups in East Azerbaijan (March 2006–March 2016)

	Elderly	Other ages	Total	*P*-value*
Head trauma	778 (57.4%)	5301 (65.9%)	7606 (81%)	<0.01
Multiple fractures	223 (16.5%)	936 (11.6%)	593 (6.3%)	
Bleeding	86 (6.3%)	610 (7.6%)	3080 (32.8%)	
Burns	4 (0.3%)	109 (1.4%)	190 (2%)	
Mixed causes	209 (15.4%)	910 (11.3%)	791 (8.4%)	
Other causes	31 (2.3%)	109 (1.4%)	661 (7%)	
Not defined	26 (1.8%)	64 (0.8%)	922 (9.8%)	
Total	1357 (100%)	8039 (100%)	9396

Ambulance was the main mode of transport for transferring the injured victims in 80% of the cases both for elderly and other age groups. This was the common pattern during the last six years, whereas, previously ambulance had had a small share of transporting the traffic injury victims.

## Discussion

In the present study, we have presented the patterns and substantial differences in traffic injury fatalities between the elderly victims and fatalities among other age groups.

Pedestrians comprised a large majority of the elderly victims and nearly two-thirds of the accidents were a vehicle-pedestrian crash, while in other age groups, a vehicle-vehicle crash was the most common accident mechanism. Vulnerable road users including pedestrians account for half of all road traffic deaths globally [9]. In the present study, more than 60% of the elderly victims were pedestrians in contrast with other age groups who were mostly vehicle occupants. Previous studies in US have found that death among the elderly mainly happened to motor vehicle occupants rather than pedestrians [10–12].We believe this may largely be due to a higher proportion of the elderly being able and willing to drive in American vs. the Iranian population. This rate may either increase in future decades in Iran. However, other explanations should also be considered such as a lower level of elderly-oriented traffic safety thinking in Iran or lower quality of medical care delivered for the elderly, and the lower coverage of EMS transfer for inner city areas and pedestrians. The proportion of road traffic deaths among vulnerable road users is greater in low- and middle-income countries than in high-income countries [9]. Older Australian pedestrians are shown to represent 13% of the population while accounting for 36% of the mortalities [12]. The elderly comprise less than 10% of the population of East Azerbaijan Province and comparing this figure with the proportion of elderly fatalities, it can be seen that the elderly are at a higher risk of traffic mortality even without considering the hypothesis that they are less frequently in the traffic environment than others. Bhalla et al., in a study in Iran, similarly showed that the elderly are 2.6 times more likely than other age groups to die due to traffic injuries [5]. Similarly, a Chinese study showed that pedestrian injuries comprise about 80% of elderly traffic injuries [13].

Pedestrians are at high-risk in vehicle impacts and the likelihood of fatality is intensified among the elderly; firstly because they are at higher risk of being involved in a crash, potentially due to their physical vulnerabilities as well as their potentially impaired cognitive capabilities; secondly due to their co-morbidities and poor physical capacity to tolerate traumas as well as the greater susceptibility of the elderly to metabolic, surgical, and infectious complications after admission to hospital [14]. Moreover, the appropriateness and quality of the delivered care also needs to be considered among the factors affecting traffic injuries mortality in the elderly. Ageism can be a concern with respect to elderly people needing medical care and trauma care is not an exception [15]. It has also been shown that triage criteria may fail to identify geriatric patients with trauma [16, 17]. Although, in the present study, head injuries were the most common type of injury regardless of the age group, the elderly who died had a lower percentage of head injuries and a higher percentage of injuries to the torso, pelvis and limbs compared to younger victims. The same pattern existed for the main cause of death being head trauma in both groups but with higher percentages of death due to multiple fractures among the elderly. Bumper collisions might explain for the higher proportion of limb fractures and potential collisions with windshields and hoods should be taken in account for upper body injuries other than those caused by violent impact with the road surface [18–20]. Moreover, it should also be taken into account that injury patterns differ between fatally injured older people and traffic fatalities of other ages in other countries. A recent study in Germany showed that the elderly who die at the scene of the accident more often show serious thorax injuries and pelvic fractures than those of other ages [21]. According to an Egyptian study, while head trauma and limb injuries were the most common type of organ injuries among pedestrians, thorax injuries were more common among car accidents where the car users were elderly [22].

In the present study, about 56% of the elderly vs. 39% in other age groups had died in hospital in East Azerbaijan province of Iran. A similar pattern is reported to exist in a study on fatal traffic injuries among pedestrians, cyclists and motor vehicle occupants in Romania [23] .The elderly deaths were twice as likely to happen at the hospital compared to fatalities in other age groups. Several explanations could be considered for this including: elderly traffic injuries are more common in inner city accidents such that they have a higher likelihood of getting to hospital in a shorter time and are as a result less likely to die before getting there possibly due to shorter distance from the accident scene to the hospital in inner city crashes compared to outer city crashes. Another explanation could be that some elderly die due to co-morbidities making them more liable to die from the trauma complications that usually happen at the hospital.

Although motor vehicle occupants comprise the majority of traffic-related head traumas, fatality among pedestrians with severe brain injury is higher than motor vehicle occupants [23]. A good emergency medical service is crucial in efforts to decrease traffic mortality through providing: on-scene emergency care, in-transit medical care, and rapid transport of the victims to hospital to receive definitive care. As found in this study, the rate of victims transferred by EMS has sharply increased after 2009. This was the year when the Iranian Ministry of Health greatly improved its EMS facilities. A sevenfold increase in the number of ambulances, fourfold increase in EMS posts and substantial decrease in transport time should not be ignored when considering decreasing traffic fatality rates during recent years [24, 25]. However, the results warrant a review of trauma system management and available facilities to ensure correct procedures and equipment is available to accurately triage and treat elderly traffic victims [26].

Unfortunately, very few Iranian studies have focused on traffic injuries among the elderly. The study by Bhalla et al., as cited earlier, is not specifically conducted on the elderly population but provides useful information on some aspects of elderly traffic injuries in Iran [5]. One mostly hospital-based study has been conducted specifically on the elderly population in Guilan province of Iran [27]. In a cross-sectional study, pre-hospital emergency system reports, hospital and police records of all motor vehicle collisions involving injured over 60 years old admitted to Pour-Sina hospital in Guilan Province between 2011 and 2012 were investigated. Although our study was based on fatalities and forensic medicine information, there were similarities between the results of the current study and the study from Guilan, insofar as the mean age of patients in the Guilan study was about 71. Most of the cases were male (74.7%). Pedestrians, with 40.5%, comprised the majority of fatalities in the Guilan study while in our study, the figure reached up to about 61%. This difference can at least partially reflect the difference in study populations (hospital data versus forensic data) and attributed to a higher fatality rate of pedestrian injuries. A second explanation may be that motorcycles are a popular mode of transportation in Guilan Province which is both a touristic and agricultural area in Iran. Actually, we do not have enough evidence to compare the proportion of motorcycles used in Guilan with East Azerbaijan, but a high percentage of elderly injuries, up to 19% in Guilan study vs. 6% in present study, were motorcycle users.

Providing a safer environment for the elderly should focus on pedestrian safety both in inner city and outer city areas. Moreover, providing service for pedestrians, fortifying urban EMS systems and improving hospital care is another field of intervention, mostly for the health sector, to reduce the burden of traffic fatalities among the elderly [28]. Without doubt, the car industry should also contribute to the work in decreasing the elderly traffic fatalities. This can be achieved by taking advantage of the latest innovations in safety technology, such as pedestrian detection systems, and thereby producing vehicles which are safer for other road users.

### Limitations

Although the data collection tools were prepared by national level experts and tested over years in the Iranian forensic medicine organization, the validity and reliability statistics for them are not published yet. However, details of classification and categorizations are recently published (8). Further details of the injuries leading to death such as classifications of intracranial hemorrhage or spine injuries or details to investigate bumper injuries are not consistently registered by forensic physicians and are not provided in the present study. That is to say some physicians do not report such details. Although such details are not usually provided in most published epidemiological studies, we recommend the national Forensic Medicine Organization authorities to add such information to the current registry as obligatory information. However, the general pattern of injuries provided could also be beneficial in understanding the epidemiology of fatalities.

## Conclusions

Some important epidemiologic features of fatal injuries such as predominance of pedestrian fatalities, pre-hospital mortality ratio and distribution of counterpart vehicles involved in crash are different among the elderly from traffic fatalities in other ages in East Azerbaijan. Pedestrian inner city crashes are a major cause of road injury fatalities among the elderly and should be considered as a priority in road safety interventions in East Azerbaijan.
